# Cerebrospinal Fluid and Serum d-Serine Levels in Patients with Alzheimer’s Disease: A Systematic Review and Meta-Analysis

**DOI:** 10.3390/jcm9123840

**Published:** 2020-11-26

**Authors:** Chun-Hung Chang, Hsiao-Lun Kuo, Wei-Fen Ma, Hsin-Chi Tsai

**Affiliations:** 1 Institute of Clinical Medical Science, China Medical University, Taichung 40402, Taiwan; chang763@gmail.com; 2 Department of Psychiatry & Brain Disease Research Center, China Medical University Hospital, Taichung 40402, Taiwan; 3 An Nan Hospital, China Medical University, Tainan 709204, Taiwan; n23931@mail.tmanh.org.tw; 4 School of Nursing, China Medical University, Taichung 40402, Taiwan; lhdaisy@mail.cmu.edu.tw; 5 Ph.D Program for Health Science and Industry, China Medical University, Taichung 40402, Taiwan; 6 Adjunct Supervisor, Department of Nursing, China Medical University Hospital, Taichung 40402, Taiwan; 7 Department of Psychiatry, Tzu-Chi General Hospital, Hualien 970473, Taiwan; 8 Institute of Medical Sciences, Tzu Chi University, Hualien 970473, Taiwan

**Keywords:** d-serine, Alzheimer’s disease

## Abstract

Objective: Alzheimer’s disease (AD) is a complex and severe neurodegenerative disease and still lacks effective methods of diagnosis. Dysfunction of the N-methyl-D-aspartate receptor (NMDAR) has been found to be involved in synapse dysfunction and neurotoxicity of AD mechanisms. d-Serine, an NMDAR receptor coagonist, is reported as a potential new biomarker for AD. However, the results of serum and cerebrospinal fluid (CSF) d-serine levels are conflicting. We conducted a meta-analysis to investigate the serum and CSF d-serine levels in patients with AD. Methods: We searched PubMed, the Cochrane central register of controlled trials, and the Cochrane database of systematic reviews for trials that measured d-serine levels both in patients with AD and in controls. We included controlled trials that analyzed d-serine levels in human samples (e.g., serum and CSF). Studies were pooled using a random-effect model for comparisons between AD and control group. We used effect size (ES; expressed as d-serine levels) in each selected meta-analysis to calculate standardized mean difference (SMD). Positive values indicated increased d-serine levels in AD group. We presented results with 95% confidence intervals (CIs). The heterogeneity of the included trials was evaluated through visually inspecting funnel plots and using the I^2^ statistic. Moderators of effects were explored using metaregression. Results: Seven trials with more than 1186 participants were included in this meta-analysis. d-serine levels in patients with AD were significantly higher than those in controls (SMD = 0.679, 95% CI = 0.335 to 1.022, *p* < 0.001). Subgroup analyses showed that the AD group had significantly higher d-serine levels in serum and CSF compared with the control group (SMD = 0.566 (serum) and 1.008 (CSF); 95% CI = 0.183 to 0.948 (serum) and 0.168 to 1.849 (CSF)). Moreover, a metaregression revealed a significant negative association between ES and mean mini-mental state examination score in AD group (slope = −0.1203, *p* = 0.0004). Conclusions: Our results revealed higher d-serine levels in the serum and CSF of patients with AD relative to the controls. Further studies with a larger sample size and longer follow-up are recommended to clarify this association.

## 1. Introduction

Alzheimer’s disease (AD) is a complex and incurable neurodegenerative disease and the most common cause of dementia. It is characterized by progressive memory loss and cognitive impairment [1]. Deposition of amyloid plaques, neurofibrillary tangles, and significant synapse loss are observed in brain pathology in patients with AD [2]. Even though evidence shows that accumulation of amyloid-β oligomers (AβOs) in brains results in synapse failure and memory loss, the precise mechanisms of AD still remain unclear.

The current diagnostic methods of AD rely on cognitive tests, imaging techniques, and cerebrospinal fluid (CSF) assays [3,4]. Currently, diagnostic guidelines include cerebrospinal fluid (CSF) levels of amyloid-β1-42 (Aβ42), total tau protein, and hyperphosphorylated tau (p-tau) [5,6]. CSF biomarkers like Aβ42 and p-tau have been used for research purposes. However, the available methods are expensive and relatively invasive [7,8]. Moreover, sensitivity and specificity of CSF Aβ42 and p-tau biomarkers have raised concerns about their clinical implication [5,9,10]. The sensitivity of CSF Aβ42 ranges from 0.69 to 0.81 and specificity ranges from 0.44 to 0.89 [11]. In addition, patients with AD are generally diagnosed late. If AD can be detected in early stages before major brain damage develops, patients may benefit more from treatment. There is an important need to develop biomarkers which can detect AD onset or at early stages. Biomarkers can improve diagnostics and allow treatment to be initiated at the earliest possible stage [12].

Abnormal hyperfunction of the N-methyl-D-aspartate receptor (NMDAR) has been found to be involved in synapse dysfunction and neurotoxicity of AD mechanisms [13,14,15,16]. Based on these mechanisms, memantine, an NMDAR partial antagonist, has been approved for patients with moderate to severe AD [17]. d-serine, one of major coagonists at the NMDARs, has been found to be related with NMDAR-mediated neurotoxicity [18,19,20]. On the other hand, d-serine has been shown to increase adult hippocampal neurogenesis [21]. In animal models, Aβ oligomers may upregulate serine racemase (SR) and thereafter increase the d-serine levels [22]. In β-amyloid 1–42 injected mice, d-serine improves motor and cognitive impairments by the inhibiting the c-Jun N-terminal kinase (JNK) signaling pathway [23].

Whether d-serine levels are altered in patients with AD remains controversial. Several trials investigated CSF d-serine levels. Two studies reported that CSF d-serine levels in an AD group were significantly higher than in a control group [22,24]. However, other trials revealed no significant difference in such levels between patients with AD and controls [25,26]. In recent years, some researchers measured d-serine levels in peripheral serum. Obtaining samples from peripheral blood is less invasive than CSF. They observed that d-serine levels in an AD group were significantly higher than those in a control group [27,28]. However, one study [29] revealed that serum d-serine levels in the AD group were lower than in normal controls, but not significantly.

Due to the inconsistency associated with serum d-serine levels in AD patients compared to controls, we conducted a meta-analysis to investigate the serum and CSF d-serine levels in patients with AD.

## 2. Methods

We followed the preferred reporting items for systematic reviews and meta-analyses (PRISMA) guidelines [30]. This study was approved by the Institutional Review Board of China Medical University, Taiwan (CMUH108-REC-006).

### 2.1. Search Strategy and Inclusion Criteria

**Figure 1:**
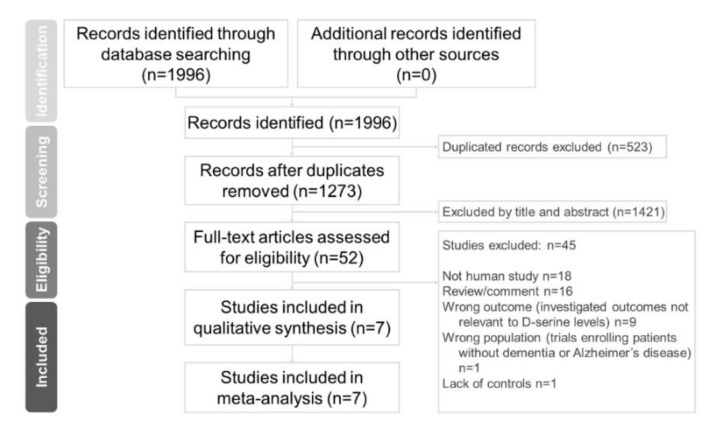
Preferred reporting items for systematic reviews and meta-analyses (PRISMA) flow diagram for searching and identifying included studies. Database: PubMed (*n* = 1996), Cochrane central register of controlled trials (*n* = 721), and Cochrane database of systematic reviews (*n* = 2). Keywords: (d-serine OR D-amino acid* OR biomarker) AND (dementia OR Alzheimer*) AND (serum OR plasma OR blood OR CSF) AND trial. Date: available until 23 November 2020.

Two independent authors (Chun-Hung Chang and Hsiao-Lun Kuo) conducted a systematic literature search from the study’s inception until 23 November 2020. We used keywords including Alzheimer’s disease, D-amino acids, and d-serine in previous trials [22,25,26,29]. We added keywords like biomarker, serum, plasma, blood, and CSF. We referred to a meta-analytic study on d-serine [31]. Finally, we extensively searched the following keywords in our search: “(d-serine OR D-amino acid* OR biomarker) AND (dementia OR Alzheimer*) AND (serum OR plasma OR blood OR CSF) AND trial)” on PubMed, the Cochrane central register of controlled trials, and the Cochrane database of systematic reviews (Figure 1). These authors (CH Chang and HL Kuo) independently evaluated the titles and abstracts of all retrieved papers for potential eligibility. We reviewed full-text articles related to potentially eligible trials and then compiled a final list of studies for inclusion. Between-reviewer discrepancies were resolved through discussion under the supervision of a third reviewer (Professor Tsai). After a thorough review of full-text articles, seven studies were ultimately included in our meta-analysis.

### 2.2. Eligibility Criteria

To be included, studies had to be (a) an observational study, either with a cohort or a cross-sectional design comparing d-serine levels in patients with AD and controls or (b) a human trial. We excluded (a) non-human studies, (b) reviews and commentaries, (c) wrong outcome (investigated outcomes not relevant to d-serine levels), (d) wrong population (trials enrolling patients without dementia or Alzheimer’s disease), and (e) did not have controls.

### 2.3. Data Extraction

Two authors (CH Chang and HL Kuo) independently extracted data of interest following the PRISMA guidelines. The primary outcome was difference in d-serine level between patients with AD and controls (calculated in terms of the standard mean difference (SMD), with the corresponding 95% confidence interval (CI) and *p* value). We defined the control group as cognitively healthy participants. We contacted the primary author of a study to request original data if data were not available from the included articles. If no relevant data regarding d-serine levels were reported in the article, we attempted to use other compatible statistical parameters (e.g., *p* value, sample size, or odds ratio) to estimate the effect size (ES) [32]. These estimated ESs were then converted and pooled into SMDs. We extracted the variables of interest, including d-serine level, mean age, gender distribution (proportion of female participants), and d-serine source.

### 2.4. Quality Assessment

We used the modified Newcastle–Ottawa scale (NOS) to determine the quality of included trials. The modified NOS was based on a version used in a meta-analysis published in the British Journal of Psychiatry in 2013. The modified NOS scores ranged from 0 to 9 [33]. If a study had a score below 5 points, it was considered to be at a high risk of bias [34,35].

### 2.5. Meta-Analysis Procedure

We used a random effects model in the meta-analysis [32]. To compare the ESs of our primary outcome, we chose the statistic of SMD with 95% CI rather than the difference in means. We did so because we presumed that different units were used in each study. Furthermore, we defined the ES to indicate higher d-serine levels in AD patients than in those without AD when the SMD value was above 0. After data from included trials were extracted, we used Comprehensive Meta-Analysis software version 3 (Biostat, Englewood, NJ, USA) to conduct our meta-analyses. Statistical significance was indicated by a two-tailed *p* value < 0.05.

### 2.6. Heterogeneity and Publication Bias

In this study, we used the I^2^ statistic to evaluate study heterogeneity [36]. We evaluated heterogeneity using the Cochran Q test and its corresponding *p* value [37]. Publication bias was assessed using funnel plots [38] and Egger’s regression test [39]. If publication bias was observed, Duval and Tweedie’s trim-and-fill procedure, which is a validated model for ES estimation, was used [40].

### 2.7. Sensitivity Test

We removed studies from the meta-analysis one by one to verify that the meta-analysis results were not due to outliers within the included studies.

### 2.8. Subgroup Meta-Analysis and Metaregression

We performed subgroup analyses. In these analyses, samples were segmented by d-serine source (e.g., serum or CSF), mean age range, or AD diagnostic criteria. We conducted subgroup analyses when at least two independent data sets were available [41]. Furthermore, metaregression analyses were performed for each potential moderator.

## 3. Results

### 3.1. Characteristics of Included Studies

Seven articles met the inclusion criteria. These included studies are summarized in Table 1 [22,24,25,26,27,28,29]. Overall, in the included studies, the data of more than 1186 participants were analyzed. The average number of participants in each study was 169.43 ± 153.91 (range: 32–397), average proportion of female participants in these trials was 58.97% ± 9.53% (range: 43.60–72.30%), and average age of participants was 71.10 ± 12.11 years. Two sources of d-serine, namely serum and CSF, were used in these trials. Four studies compared serum d-serine levels in AD versus control group [25,27,28,29], and four studies compared CSF d-serine levels in AD versus control group [22,24,25,26]. One study reported d-serine levels in both serum and CSF, and we considered that study as two studies in our analysis (Serum: AD vs. Controls; CSF: AD vs. Controls) [25].

### 3.2. Meta-Analyses of Overall D-Serine Levels

**Figure 2:**
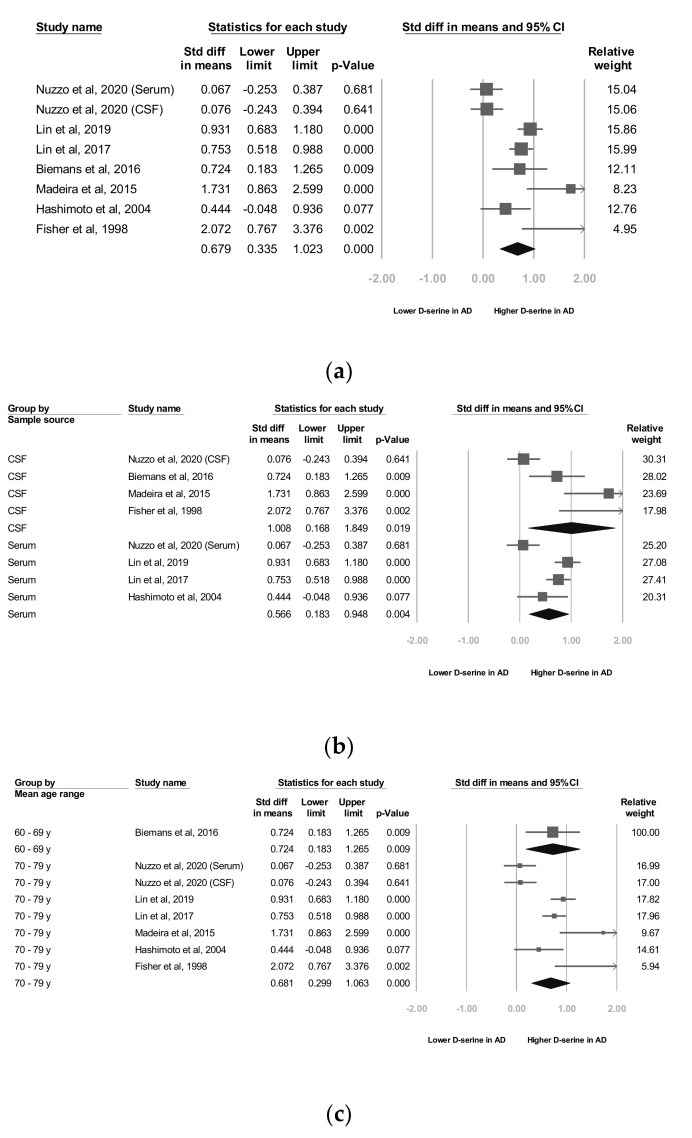

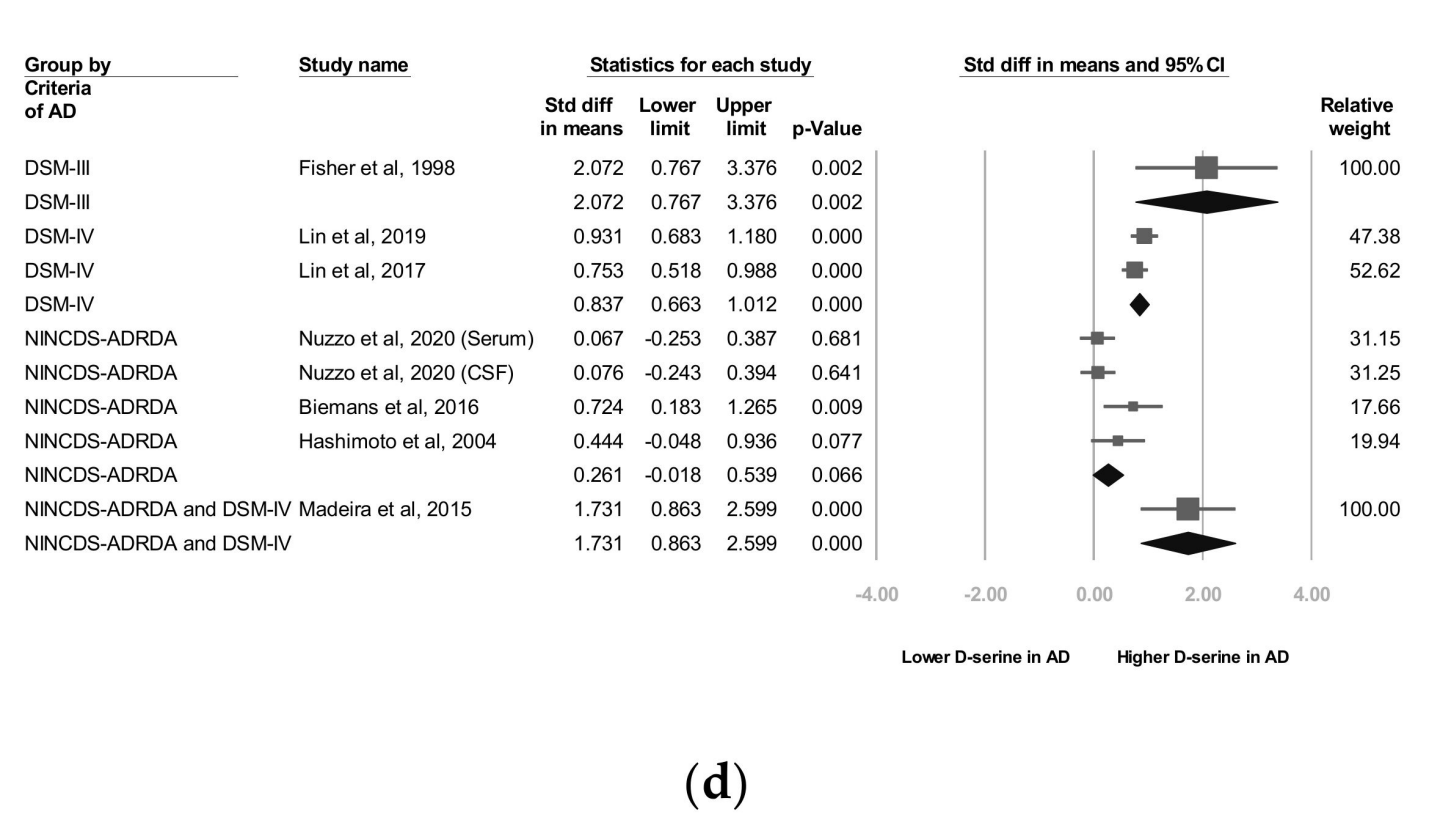
Meta-analyses of (**a**) d-serine levels in patients with Alzheimer’s disease and in controls, (**b**) subgroups segmented by d-serine source, (**c**) subgroups segmented by mean age range, and (**d**) subgroups segmented by Alzheimer’s disease criteria. (Note: in the graph, the square represents the effect size of each study. The bigger the square, the more participants in the study. A horizontal line represents the 95% confidence intervals of the study result, with each end of the line representing the boundaries of the confidence interval. The diamond represents the combined effect).

Seven studies [22,24,25,26,27,28,29] reported raw data, which we used for our meta-analyses. Our meta-analysis revealed that the d-serine levels of patients diagnosed as having AD were significantly higher than those of controls (SMD = 0.679, 95% CI = 0.335 to 1.022, *p* < 0.001; Figure 2a).

### 3.3. Subgroup Analyses of Overall D-Serine-Level Moderators

We performed several subgroup analyses, where samples were segmented by d-serine source, mean age range, and AD diagnostic criteria.

### 3.4. A Subgroup Analyses by D-Serine Source

As stated, two d-serine sources (serum and CSF) were used in these trials. The subgroup analyses revealed that d-serine levels from both serum and CSF in AD group were significantly higher than those in control group (SMD = 0.566 (serum) and 1.008 (CSF); 95% CI = 0.183 to 0.948 (serum) and 0.168 to 1.849 (CSF)). Larger ESs were observed in the d-serine level for CSF than for serum (Figure 2b).

### 3.5. B Subgroup Analyses by Mean Age Range

In six trials that enrolled patients with a mean age of 70 to 79 years, d-serine levels were significantly higher in AD group than in control group (SMD = 0.681, 95% CI = 0.299 to 1.063, *p* < 0.001). A significant and positive effect was noted in one trial in which patients with a mean age of 60 to 69 years were enrolled (SMD = 0.724, 95% CI = 0.183 to 1.205, *p* = 0.009; Figure 2c).

### 3.6. C Subgroup Analyses by AD Criteria

In two studies, codes from the Diagnostic and Statistical Manual of Mental Disorders, Fourth Edition (DSM-IV) were used as diagnostic criteria [27,28] and in three trials, the NINCDS-ADRDA Alzheimer’s Criteria were used as diagnostic criteria [25,26,29]. In one study [22], both the NINCDS-ADRDA Alzheimer’s Criteria and DSM-IV codes were employed as diagnostic criteria. The subgroup analyses showed that d-serine levels in AD group diagnosed using DSM-IV codes were significantly higher than those in control group (SMD = 0.837, 95% CI = 0.663 to 1.012, *p* < 0.001). d-serine levels in AD group diagnosed using the NINCDS-ADRDA Alzheimer’s Criteria were higher than those in control group, but non-significantly (SMD = 0.261, 95% CI = −0.018 to 0.539, *p* = 0.066; Figure 2d).

### 3.7. Metaregression Analyses of D-Serine Levels

**Figure 3:**
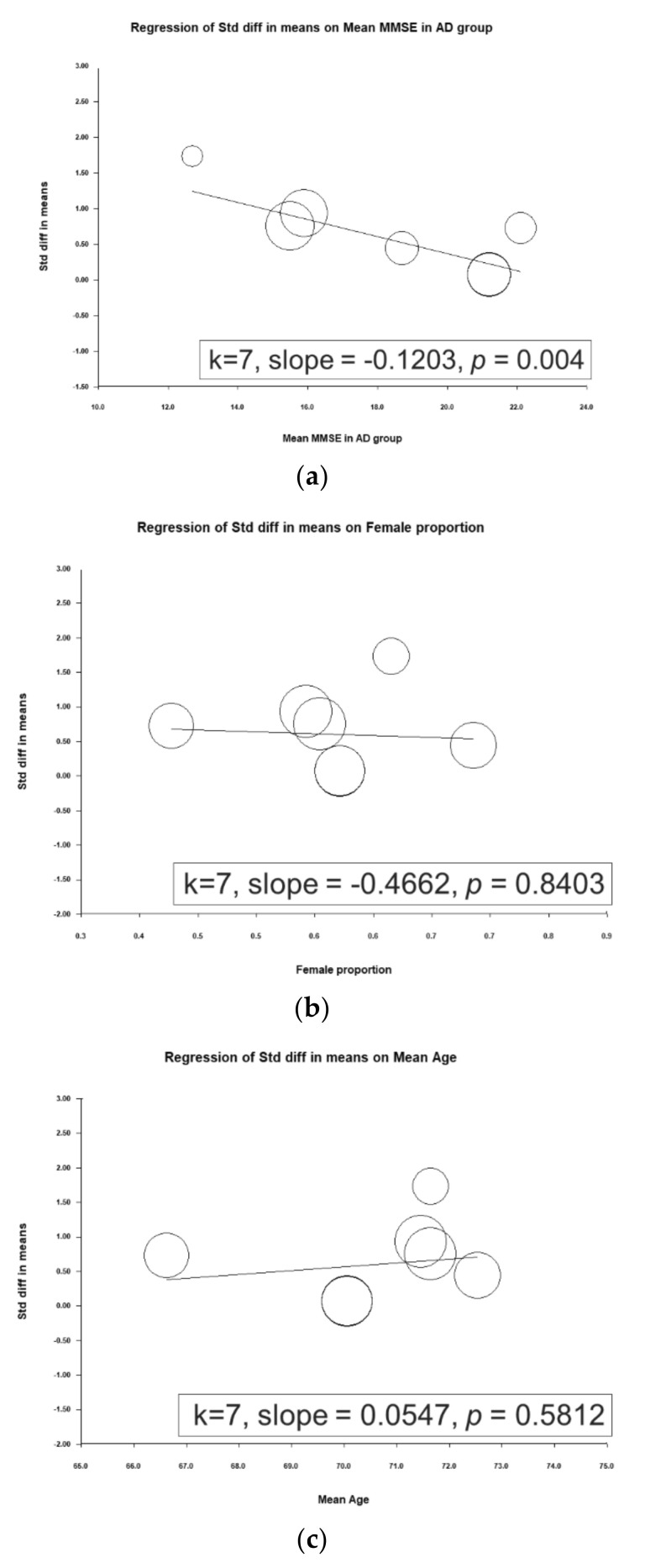
Meta-regression of the effects of d-serine levels in relation to (**a**) mean mini-mental state examination (MMSE) score in the Alzheimer’s disease group, (**b**) female sex proportion, and (**c**) mean age. (Note: in the graph, each study is represented by a circle that shows the actual coordinates (observed effect size by latitude) for that study. The size (specifically, the area) of each circle is proportional to that study’s weight in the analysis. k is the number of studies.)

A metaregression was performed to determine associations between ESs and mean mini-mental state examination (MMSE) scores in the AD group, proportion of female participants, and mean patient age. A significant inverse association was observed between the ES and mean MMSE score in the AD group (slope = −0.1203, *p* = 0.0004), meaning that a higher mean MMSE score in the AD group entailed a smaller ES (i.e., a smaller difference in d-serine levels was noted between patients with AD and controls; Figure 3).

### 3.8. Heterogeneity and Publication Bias

**Figure 4:**
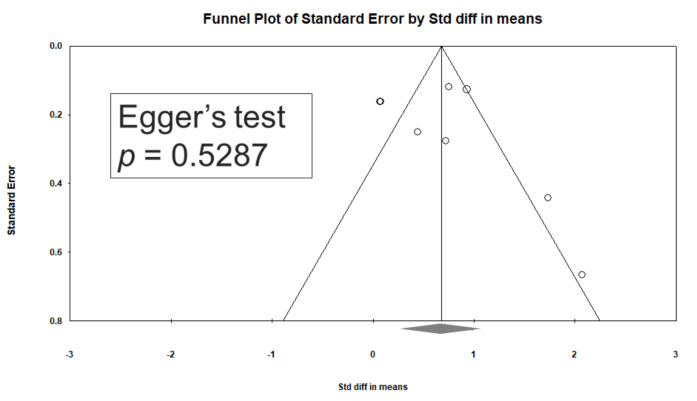
Funnel plots of d-serine levels. (Note: in the graph, the observed studies are shown as open circles, and the observed point estimate in log units is shown as a diamond.).

Significant heterogeneity was observed within these studies (Q = 41.543, df = 7, I^2^ = 83.150%, *p* < 0.001). Egger’s test revealed no significant publication bias regarding the overall SMD (*p* = 0.5287). The funnel plots for the SMD of overall cognitive function are presented in Figure 4.

### 3.9. Sensitivity Analysis

In a meta-analysis of overall d-serine levels, the results remained significant when any individual study was removed from the analysis.

## 4. Discussion

To our knowledge, this is the first meta-analysis to focus on D-serine levels in patients with AD. The main findings are as follows. (1) d-serine levels in patients diagnosed as having AD were significantly higher than those in controls (SMD = 0.679, 95% CI = 0.335 to 1.022, *p* < 0.001). (2) In subgroup analyses, d-serine levels from serum and CSF in AD group were significantly higher than those in control group (SMD = 0.566 (serum) and 1.008 (CSF); 95% CI = 0.183 to 0.948 (serum) and 0.168 to 1.849 (CSF)). (3) A significant inverse association was observed between the ES and mean MMSE score in the AD group (slope = −0.1203, *p* = 0.0004).

These findings agree with those of most related studies. Four of the studies reported significantly higher d-serine levels in patients with AD than in controls [22,24,27,28]. Two studies did not report significant differences between d-serine levels between these groups [25,26]. Furthermore, contradictory findings were obtained by Hashimoto and colleagues [29]. In their study, serum d-serine levels in AD group were lower than in normal controls, but the difference was non-significant (AD group: 1.88 ± 0.51 µM, control group: 2.14 ± 0.65 µM (*p* = 0.078)). In the work of Hashimoto and colleagues, this discrepancy may have been due to several factors. First, AD severity may be associated with d-serine level. Trials with lower MMSE scores tended to report significant differences. For example, in Lin’s study, the mean MMSE score in the AD group was 15.9 ± 4.3. However, trials in which participants had high MMSE scores tended to not report significant differences. In Nuzzo’s, the mean MMSE score in AD group was 21.2 ± 3.8. In our metaregression, a significant inverse association between ES and mean MMSE score in AD group was noted (slope = −0.1203, *p* = 0.0004), meaning that the higher the mean MMSE score in AD group was, the weaker the ES (i.e., a smaller difference in d-serine level was observed between patients with AD and controls; Figure 3). Second, another possible factor is the small sample size (32 AD patients and 33 cognitive health controls) compared to 397 participants in Lin’s study [28].

Subgroup analyses revealed that d-serine levels from serum and CSF in AD group were significantly higher than those in control group (SMD = 0.566 (serum) and 1.008 (CSF); 95% CI = 0.183 to 0.948 (serum) and 0.168 to 1.849 (serum)). d-serine in CSF was related to larger ESs (Figure 2b). In our meta-analysis, four included trials reported d-serine levels in CSF [22,24,25,26]. Two of the four trials showed that d-serine levels in AD group were significantly higher than those in controls [22,24]. Madeira and colleagues assessed d-serine levels in the CSF of 21 patients with probable AD and compared them with those in 10 healthy controls; such levels were significantly higher in the probable AD group than in controls (12.32 ± 0.44 vs. 2.45 ± 0.65, *p* = 0.0001) [22]. Another study consisting of 29 patients with AD and 28 controls without dementia revealed that D-serine levels in the AD group were significantly higher than those in controls (1.56 ± 0.29 vs. 1.35 ± 0.29, *p* = 0.03). Fisher and colleagues reported that free d-serine levels were significantly higher (*p* < 0.05) in AD ventricular CSF than in normal CSF [24]. In two of the four included studies, no significant difference was observed [25,26].

Four of the included trials reported d-serine levels in peripheral serum [25,27,28,29], two of which revealed that d-serine levels in AD group were significantly higher than in controls [27,28]. Moreover, in both these studies, d-serine levels were revealed to increase with AD severity. For example, in Lin’s study [27], the mean d-serine level in serum was 49.2 ± 27.2 ng/mL in the mid AD group, 54.0 ± 26.9 ng/mL in the moderate AD group, and 60.8 ± 25.8 ng/mL in the severe AD group. One study [29] revealed that serum d-serine levels in AD group were lower than in normal controls, but the difference was not significant (AD group: 1.88 ± 0.51 μM, control group: 2.14 ± 0.65 μM, *p* = 0.078).

Three diagnostic criteria including DSM-III, DSM-IV, and NINCDS-ADRDA were adopted in these studies. The subgroup analyses showed that d-serine levels in AD group diagnosed using DSM-IV codes were significantly higher than those in control group (SMD = 0.837, 95% CI = 0.663 to 1.012, *p* < 0.001). d-serine levels in AD group diagnosed using the NINCDS-ADRDA Alzheimer’s Criteria were higher than those in control group, but non-significantly (SMD = 0.261, 95% CI = −0.018 to 0.539, *p* = 0.066; Figure 2d). We observed that studies using DSM had lower mean MMSE scores. For example, in Lin’s study, the mean MMSE score in the AD group was 15.9 ± 4.3. On the other hand, studies using NINCDS-ADRDA had higher mean MMSE scores. In Nuzzo’s study, the mean MMSE score in AD group was 21.2 ± 3.8. Previous studies have reported discrepancies between different diagnostic criteria of AD in a geriatric cohort of patients with cognitive impairment [42,43]. Patients diagnosed by DSM may have lower MMSE scores than those diagnosed by NINCDS-ADRDA. Further trials may investigate the influence of different diagnostic criteria in d-serine levels studies.

In this study, we used metaregression to examine the effect of moderator variables on serum d-serine levels. We revealed a significant inverse association between ES and mean MMSE score in AD group. In six included trials, MMSE score was used to evaluate cognitive function in AD group [22,25,26,27,28,29]. Madeira and colleagues [22] reported that d-serine levels in CSF were negatively correlated with MMSE score, whereas Biemans and colleagues observed no correlation between d-serine levels in CSF and MMSE scores in the entire cohort (*r* = 0.25, *p* = 0.11) [26]. A recent study enrolled 144 patients; 20, 85, 25, and 14 had amnestic mild cognitive impairment (MCI), mild AD, moderate AD, and severe AD, respectively. The researchers of that study used the Alzheimer’s Disease Assessment Scale-Cognitive Subscale (ADAS-Cog) to assess cognitive function in patients with AD. They found that total ADAS-Cog score was positively correlated with both d-serine level (*r* = 0.186, *p* = 0.026) and d-serine/total serine ratio (*r* = 0.191, *p* = 0.022). Their findings indicate that D-amino acids in serum could be correlated with ADAS-Cog score in different items and play diverse roles in AD pathology [44].

Why and how increased d-serine levels involved in cognitive impairment in patients with AD is still unclear. Studies have found that d-serine may promote synaptogenesis and have memory-enhancing effects [45,46]. On the other hand, excessive d-serine levels due to increased expression of serine racemase may contribute to neuronal apoptosis [47]. Therefore, maintaining proper d-serine levels may benefit the synaptic homeostasis and function. Further studies are needed to explore the role of d-serine in synapse dysfunction and neurotoxicity in AD. The association between d-serine levels and cognitive function warrants further investigation.

## 5. Strengths

Our study had several merits. First, ours is the first meta-analysis to focus on d-serine levels in patients with AD. Second, we revealed that d-serine levels in the serum, or CSF of patients diagnosed as having AD were significantly higher than those in controls. Third, we noted a significant inverse association between ESs and mean MMSE scores in AD group.

## 6. Implications

In this meta-analytic study, we found that d-serine levels in patients diagnosed as having AD were significantly higher than those in controls. A significant inverse association was observed between the ES and mean MMSE score in the AD group. Trials with lower MMSE scores tended to report significant differences. Our results suggest that peripheral d-serine levels may be a biomarker to predict AD. However, our findings did not support d-serine as a good biomarker of MCI or pre-AD because trials enrolling participants with high MMSE scores tend to not report significant differences. Further studies with a larger sample size and longer follow-up are required to evaluate the relationship between d-serine levels and the progression of cognitive impairment. Brain imaging may be helpful for investigating the association between d-serine levels in various brain regions and d-serine levels in CSF and serum.

## 7. Limitations

This study had some limitations. First, we could observe related phenomena but not clarify the underlying pathophysiology because of the inherent methodological limitations of meta-analyses. Definitive conclusions should be drawn with caution. Second, significant heterogeneity was detected among the investigated meta-analyses. Third, measurements of d-serine levels in these included trials may not have been conducted using the same equipment and processes. Fourth, various factors in the included trials may have affected serum d-serine levels. However, we could not adjust for this comprehensive set of factors in our meta-analysis owing to a lack of further data on, for example, dietary patterns, additional nutritional supplements, subtypes of AD, medication, and the time at which the sample was measured. Therefore, further studies with a larger sample size and longer follow-up should evaluate these factors.

## 8. Conclusions

We revealed that d-serine levels from serum and CSF in AD group were significantly higher than those in control group. We also noted a significant inverse association between ESs and mean MMSE scores in AD group. Further studies with a larger sample size and longer follow-up should evaluate these factors. Advanced brain imaging may be helpful for investigating the association between d-serine levels in different brain regions and in CSF and serum.

## Figures and Tables

**Table 1:** Summary of characteristics of studies in current meta-analysis.

Study (First Author, Year)	Criteria of AD	Diagnosis	Sample Source	Comparison	Subject	Mean Age (y)	Female (%)	Cognitive Measures	Mean MMSE in AD Group	Findings
Nuzzo et al., 2020 [25]	NINCDS-ADRDA	AD	Serum and CSF	AD Control	166	70.1 (7.6)	59.6	CDR, MMSE	AD: 21.2 (3.8) (pre-AD: 27.3 (0.9) AD-MCI: 23.7 (3.1) AD-Dem: 16.0 (5.1))	No statistically significant differences
Lin et al., 2019 [27]	DSM-IV	AD	Serum	AD Control	376	71.5 (8.5)	56.4	CDR, MMSE	AD: 15.9 (4.3) (Mild AD: 18.9 (4.4) Moderate AD: 11.5 (3.7) Severe AD: 8.1 (4.2))	Higher in AD group
Lin et al., 2017 [28]	DSM-IV	AD	Serum	AD Control	397	71.6 (8.6)	57.7	CDR, MMSE	AD: 15.4 (4.5) (Mild AD: 18.5 (4.4) Moderate to severe AD: 10.4(4.6))	Higher in AD group
Biemans et al., 2016 [26]	NINCDS-ADRDA	AD	CSF	AD Control	79	66.9 (7.4)	43.6	MMSE	AD: 22.1 (3.3)	No statistically significant differences
Madeira et al., 2015 [22]	NINCDS-ADRDA and DSM-IV	AD	CSF	AD Control	71	71.7 (7.8)	64.5	MMSE	AD: 12.7 (6.2)	Higher in AD group
Hashimoto et al., 2004 [29]	NINCDS-ADRDA	AD	Serum	AD Control	65	72.5 (6.1)	72.3	MMSE	AD: 18.7 (5.0)	No statistically significant differences
Fisher et al., 1998 [24]	DSM-III	AD	CSF	AD Control	32	73.4 (9.6)	NA	NA	NA	Higher in AD group

Abbreviations: AD, Alzheimer’s disease; CDR, Clinical Dementia Rating; CSF, cerebrospinal fluid; DSM-III, Diagnostic and Statistical Manual of Mental Disorders, Third Edition; DSM-IV, Diagnostic and Statistical Manual of Mental Disorders, Fourth Edition; MMSE, Mini-Mental State Examination; NA, not available; NINCDS-ADRDA, National Institute of Neurological and Communicative Disorders and Stroke and the Alzheimer’s Disease and Related Disorders Association criteria; Pre-AD, pre-clinical Alzheimer’s disease; AD-MCI, Alzheimer’s disease–related mild cognitive impairment; AD-Dem, Alzheimer’s disease dementia.
